# Proteomics Analysis Reveals Bacterial Antibiotics Resistance Mechanism Mediated by *ahslyA* Against Enoxacin in *Aeromonas hydrophila*

**DOI:** 10.3389/fmicb.2021.699415

**Published:** 2021-06-08

**Authors:** Zhen Li, Lishan Zhang, Qingli Song, Guibin Wang, Wenxiao Yang, Huamei Tang, Ramanathan Srinivasan, Ling Lin, Xiangmin Lin

**Affiliations:** ^1^Fujian Provincial Key Laboratory of Agroecological Processing and Safety Monitoring, School of Life Sciences, Fujian Agriculture and Forestry University, Fuzhou, China; ^2^Zhangzhou Health Vocational College, Zhangzhou, China; ^3^Key Laboratory of Crop Ecology and Molecular Physiology, Fujian Agriculture and Forestry University, Fujian Province University, Fuzhou, China; ^4^Key Laboratory of Marine Biotechnology of Fujian Province, Institute of Oceanology, Fujian Agriculture and Forestry University, Fuzhou, China; ^5^State Key Laboratory of Proteomics, Beijing Proteome Research Center, National Center for Protein Sciences, Beijing Institute of Lifeomics, Beijing, China

**Keywords:** antibiotics resistance, AhSlyA, enoxacin, quantitative proteomics, *Aeromonas hydrophila*

## Abstract

Bacterial antibiotic resistance is a serious global problem; the underlying regulatory mechanisms are largely elusive. The earlier reports states that the vital role of transcriptional regulators (TRs) in bacterial antibiotic resistance. Therefore, we have investigated the role of TRs on enoxacin (ENX) resistance in *Aeromonas hydrophila* in this study. A label-free quantitative proteomics method was utilized to compare the protein profiles of the *ahslyA* knockout and wild-type *A. hydrophila* strains under ENX stress. Bioinformatics analysis showed that the deletion of *ahslyA* triggers the up-regulated expression of some vital antibiotic resistance proteins in *A. hydrophila* upon ENX stress and thereby reduce the pressure by preventing the activation of SOS repair system. Moreover, *ahslyA* directly or indirectly induced at least 11 TRs, which indicates a complicated regulatory network under ENX stress. We also deleted six selected genes in *A. hydrophila* that altered in proteomics data in order to evaluate their roles in ENX stress. Our results showed that genes such as *AHA_0655*, *narQ*, *AHA_3721*, *AHA_2114*, and *AHA_1239* are regulated by *ahslyA* and may be involved in ENX resistance. Overall, our data demonstrated the important role of *ahslyA* in ENX resistance and provided novel insights into the effects of transcriptional regulation on antibiotic resistance in bacteria.

## Introduction

Antibiotic-resistant bacterial strains were discovered over 90 years ago. Since then, antibiotic-resistant bacterial strains have been found to be widely distributed in various environments, such as in hospitals, seafood, and aquaculture farms, and as a result, they pose a serious public health problem worldwide (Fritsche et al., 2009; Peng et al., 2015). Although, several mechanisms of antibiotic resistance such as membrane permeability, plasmid transfer, antibiotic modification or degradation, efflux, and biofilm formation have been described in recent years, the underlying mechanism of acquire antibiotic resistance in bacteria is still largely unknown (Peterson and Kaur, 2018). The emergences of drugs resistant bacterial strains have been caused by many complicated characteristics, one of which is the antimicrobial resistance genes (ARGs) transcriptional regulators (TRs). Bacterial TRs play an important role in the transcriptional regulation of functional genes needed to survive environmental stresses, including antibiotic resistance (Zhou and Yang, 2006; Tang et al., 2019a). For example, a mutation in the multiple antibiotic resistance (MarR) TRs in *Escherichia coli* has been shown to lead to the expression of the *marRAB* operon. Therefore, it promotes the expression of the transcription factor MarA and activation of the *acrAB* and *tolC* efflux pump genes, resulting in multi-drug resistance to tetracycline, quinolones, β-lactams, and phenolic compounds (Pourahmad Jaktaji and Ebadi, 2013; Lankester et al., 2019). Additionally, the TR EmrR is an inhibitor of efflux pump EmrCAB in *Chromobacterium violaceum* and the mutation of *emrR*_R92H_ increases the resistance of *C. violaceum* to nalidixic acid (Barroso et al., 2018). Nonetheless, there are hundreds of bacterial TRs with biological functions that are poorly characterized and most of the direct and indirect effects of TRs on bacterial antibiotic resistance are largely unknown.

*Aeromonas hydrophila* is a widely distributed environmental bacterium and a well-known fish pathogen. The use of antibiotics in aquaculture industries has resulted in the emergence of multi-drug resistant *A. hydrophila* strains in aquaculture and even in hospital settings (Li et al., 2020; Zhu et al., 2020). In recent years, many research, including our previous study, have been found that the several metabolic pathways related genes were involved in the drug-resistance on this pathogen by multi omics technologies (Hossain et al., 2018; Sun et al., 2019; Li et al., 2021). In our previous study, we reported that the LysR-type TR YeeY in *A. hydrophila* plays an important role in the regulation of furazolidone resistance by directly regulating ARGs, including *AHA_3222* and *AHA_4275*. It indicates the crucial role of TRs in the antibiotic resistance of this pathogen. However, the underlying mechanisms of antibiotic resistance regulated by TRs in *A. hydrophila* are needed to be further investigated.

To better understanding the role and regulatory mechanism of TR on the bacterial physiological function. In this study, we reported on a MarR family TR in *A. hydrophila*, AhSlyA (gene name *ahslyA* or *AHA_1240*). AhSlyA is a winged helix-turn-helix (wHTH) DNA-binding TR. Previous research reported the homologous proteins of this TR in other bacterial species play diverse biological functions such as cell metabolism and virulence, while its biological effect and molecular mechanism are still largely unknown, especially for the bacterial antibiotics resistance in *A. hydrophila* (Banda et al., 2019; Tian et al., 2021). In this study, we constructed an *ahslyA* deletion mutant (Δ*ahslyA*) in *A. hydrophila* and found that it displayed significantly decreased resistance to the quinolone antibiotic, enoxacin (ENX), as compared to the wild-type (WT) parent strain. To further investigate the direct or indirect effect of this TR on the antibiotic resistance of *A. hydrophila*, a label-free quantitative proteomics method was used to compare the differentially expressed proteins between the Δ*ahslyA* and WT strains under ENX stress. Moreover, a several differentially expressed genes were deleted and their antibiotic susceptibility to ENX was validated. This study will conduce to further understand the complicated antibiotic resistance mechanisms mediated by bacterial TRs.

## Materials and Methods

### Bacterial Strains and Culture Conditions

The strains used in this study are *A. hydrophila* ATCC 7966, Δ*ahslyA*, and the *ahslyA* complemented strain. *E. coli* MC1061 and S17 were stored in our laboratory previously. The culture temperature of *A. hydrophila* and *E. coli* were 30 and 37°C, respectively. Both bacterial strains were cultured in Luria–Bertani (LB) medium with appropriate antibiotics.

### Construction of the Gene Deletion Strain

The deletion strain was constructed based on the principle of two-step homologous recombination using the suicide vector pRE112, as previously described (Yu et al., 2005). Briefly, the pRE112 plasmid fused with about 500-bp of the upstream and downstream flanking regions of the target gene was constructed using *A. hydrophila* ATCC 7966 genomic DNA as template and then transferred into competent *E. coli* MC1061 cells. Then, the plasmid of a positive clone was extracted and transferred into *E. coli* S17 competent cells. After verifying by PCR amplification, the *E. coli* S17 carrying the pRE112 recombined vector was then conjugated with *A. hydrophila* in a 4:1 ratio to the first step of homologous recombination. Positive *A. hydrophila* clones were selected on LB agar plates with ampicillin and chloramphenicol (Yeasen Inc., Shanghai, China). The second step of homologous recombination was carried out in LB medium containing 20% sucrose. The Δ*ahslyA* in *A. hydrophila* was confirmed by plating chloramphenicol, followed by PCR and DNA sequencing. Finally, after about 20 generations of stable inheritance and correct DNA sequencing, the Δ*ahslyA* mutant was stocked and stored in the freezer at −80°C.

### Protein Sample Preparation

Bacterial strains were inoculated in 5 mL LB medium, cultured for 16 h and then transferred at the ratio of 1:100 into 100 mL of LB medium containing ENX at a final concentration of 0.0078 μg/mL. After culturing for about 3 h (until the culture reached an OD_600_ of approximately 1.0), the cells were collected by centrifugation at 8,000 × *g* at 4°C for 20 min and washed twice with PBS. The bacterial samples were then resuspended in 5 mL of PBS buffer containing 1 mM phenylmethanesulfonyl fluoride (PMSF) and then lysed by ultra-sonication. The supernatant was collected by centrifugation at 8,000 × *g* for 20 min and the total protein concentration was detected *via* the Bradford method. About 50 μg of each protein sample was reduced with 50 mM dithiothreitol at 56°C, alkylated with 25 mM iodoacetamide in dark and then digested to peptides with a 1:20 ratio of trypsin (Promega Inc., Shanghai, China). The enzymatic peptides were desalted with a C18 column (Waters Inc., Milford, MA, United States) and dried with a centrivap concentrator (Labconco Inc., Kansas City, MO, United States). Each group sample was performed three independent repeats for biological replicates.

### Label-Free LC-MS/MS

The desalted peptides were dissolved in liquid chromatography mobile phase buffer A [containing 2% acetonitrile, 0.1% formic acid (FA)], loaded onto the pre-column at a flow rate of 4.5 μL/min on the chromatographic system and then injected into the column at a flow rate of 300 nL/min by an easy-nlc1200 system (Thermo Scientific Inc., Waltham, MA, United States). The liquid gradient setting was as follows: 0–3 min, buffer B (containing 80% acetonitrile, 0.1% FA) increased linearly from 2 to 5%; 3–103 min, solution B increased linearly from 5 to 28%; 103–108 min, solution B increased linearly from 35 to 90%; 110–120 min, the solubility of solution B was maintained at 90%. Mass spectrometry was performed with an Orbitrap Fusion Lumos system (Thermo Scientific Inc., Waltham, MA, United States) nanospray ion source. The spray voltage was 2.0 KV and the ion transfer tube temperature was 300°C. We used the data-dependent acquisition mode to the collect data. The parameters were as follows: the precursors from 350 to 1,600 m/z were scanned at a resolution of 60,000, and the AGC target was set at 4e5. For MS/MS, the HCD collision energy was 30% with a resolution of 15,000. The AGC target was set to 5e4. The cycle time was 3 s. All raw data were searched by Maxquant software v.1.6.3.4 against Uniprot *A. hydrophila* ATCC7966 database. Proteins with the number of peptides greater than 2, *p*-value less than 0.05, and protein ratio difference greater than 1.5 times were selected as differential proteins for bioinformatics analysis. The raw MS files were submitted to the iProx (Integrated Proteome resources) database under the accession number IPX0002908000 (Ma et al., 2019).

### Bioinformatics Analysis

The GO (gene ontology) analysis of altered proteins were performed using the online software DAVID^1^ and visualized with GOplot package in R language software (Walter et al., 2015; Zhang L.S. et al., 2020). The protein-protein interaction (PPI) network was predicted using the String^2^ online database with a confidence score ≥ 0.7 and the network was clustered using the “Markov Cluster Algorithm (MCL)” and the inflation parameter was set as 4 (Szklarczyk et al., 2017). Finally, the visualized network diagram of PPI was drawn using Cyctoscape 3.8.0^3^ (Shannon et al., 2003).

### Determination of Minimal Bactericidal Concentrations

The minimal bactericidal concentration (MBC) assay was performed by the agar dilution method, as previously described (Jiang et al., 2020). Briefly, an overnight bacterial culture was passaged into fresh LB medium, incubated at 30°C with shaking until the OD_600_ reached about 1.0 and then diluted 100 times. Then, 2 μL of each dilution was spotted onto an LB agar plates with twofold dilution gradient concentration of antibiotics (ciprofloxacin, levofloxacin, enoxacin, and enrofloxacin purchased from Yeasen biotech, Ltd., Shanghai, China), respectively, and incubated at 30°C for 16 h. Each experiment was performed at least three times with biological duplicates.

## Results

### *ahslyA* Mutant Affects Antibiotic Susceptibility in *Aeromonas hydrophila*

**FIGURE 1:**
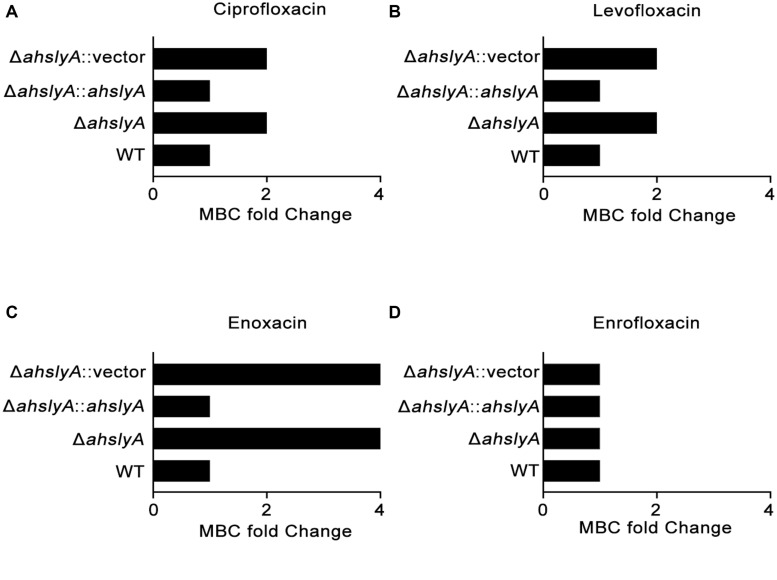
Measurement of the MBCs of *ahslyA* derivatives in *A. hydrophila* to four quinolone antibiotics. **(A)** The MBCs of Δ*ahslyA* and complemented Δ*ahslyA* strains, in addition to WT and Δ*ahslyA* carrying an empty vector as negative controls, were tested for their sensitivity to **(A)** ciprofloxacin, **(B)** levofloxacin, **(C)** enoxacin, and **(D)** enrofloxacin. The MBCs of each strain to antibiotics were compared with WT as MBC fold change.

In order to better understand the characteristics of the TR AhslyA on bacterial antibiotic resistance, we first constructed Δ*ahslyA* mutant strain and then tested its antibiotic susceptibilities against various antibiotics, including several quinolone antibiotics. As shown in Figure 1, loss of *ahslyA* caused a twofold increase in the MBC to ciprofloxacin (CIP) and levofloxacin (LVX), and increased the MBC of ENX by four times; however, it did not affect the susceptibility to enrofloxacin (ENR). Moreover, complementation of the Δ*ahslyA* strain restored the antibiotic susceptibilities similar level to the WT strain, which is suggesting that the *ahslyA* gene in *A. hydrophila is* involved in the regulation of several antibiotic resistances, especially ENX.

### Quantitative Proteomics Comparison Between WT and Δ*ahslyA* Strains Under ENX Stress

**FIGURE 2:**
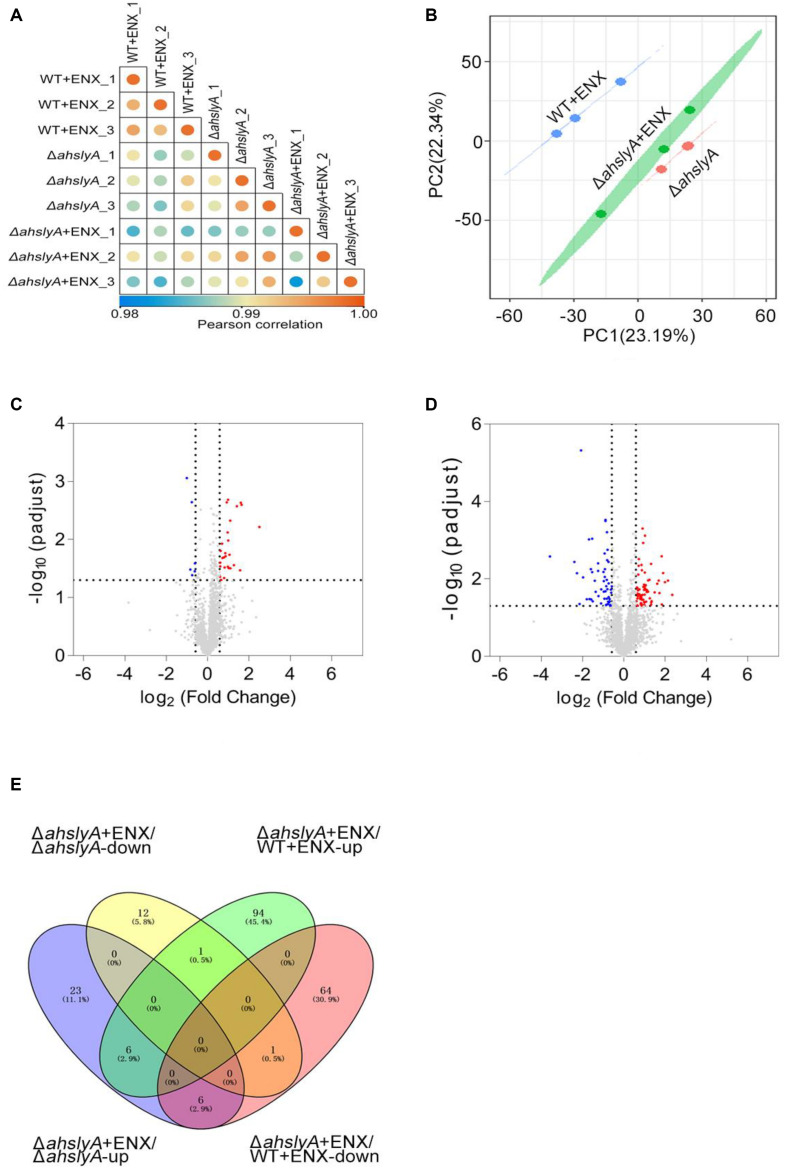
Label-free quantitative proteomics data analysis. **(A)** Correlation coefficient analysis of protein MS intensity in three biological replications. **(B)** PCA of WT + ENX, Δ*ahslyA* + ENX, and Δ*ahslyA*. **(C,D)** Volcano maps comparing the abundance ratios of proteins with significant differences in expression between Δ*ahslyA* + ENX vs. Δ*ahslyA* and Δ*ahslyA* + ENX vs. WT + ENX, respectively. The blue dots represent downregulated proteins, red dots represent upregulated proteins, and the gray color represents non-differentially expressed proteins. **(E)** Venn-diagram of differentially expressed proteins between two comparisons.

In order to further investigate the regulatory mechanism of *ahslyA* on ENX antibiotic resistance, we isolated whole protein samples from WT and Δ*ahslyA* strains with or without exposure to 0.0078 μg/mL ENX treatments. After trypsin digestion, each sample was quantified by a label-free quantitative proteomics method to compare the differentially expressed proteins between both groups. As shown in Figure 2A, positive correlations greater than 0.98 were found between the intensities of the MS of each sample with the regression coefficients, which indicated that the quantitative analysis results of MS in this study were stable and reliable. A principal component analysis (PCA) scatter diagram was drawn to cluster the samples. The dots of different colors in Figure 2B represent three groups of repeats of the same sample. It can be seen from the diagram that the three dots of the same color are relatively close; indicating that the gene expression pattern of the three repeats of the same sample had a small difference and suitable data repeatability. The proteomic analysis of the Δ*ahslyA* + ENX group was significantly separated from the WT + ENX group in the direction of PC1, indicating that there may be significant differences between them. These results indicate that the effect of the deletion of *ahslyA* on bacterial proteome is more than the effect of ENX stress.

### The Differential Protein Abundances of WT and Δ*ahslyA* in Response to ENX Stress

In this study, a total of 2,534 proteins were identified by mass spectrometry (unique peptide number ≥ 2 and false discovery rate (FDR) < 1%). The protein abundance ratio that was ≥ 1.5 (upregulated expression) and ≤0.667 (downregulated expression) with a *p*-value < 0.05 of each compared group was regarded as differentially expressed proteins. We analyzed the data by two comparisons, Δ*ahslyA* + ENX vs. Δ*ahslyA* and Δ*ahslyA* + ENX vs. WT + ENX, in order to interpret the *ahslyA* mediated ENX resistance in this study. When compared with Δ*ahslyA* without antibiotic treatment, there was 49 differentially expressed proteins were found, which accounted for 1.77% of the total identified proteins, including 35 increasing and 14 decreasing in abundance in the Δ*ahslyA* + ENX treatment group (Figure 2C). When compared with WT + ENX, a total of 172 proteins, including 101 increasing and 71 decreasing in abundance, were altered in the Δ*ahslyA* + ENX treatment group (Figure 2D). The following overlap analysis between both group comparisons showed that Δ*ahslyA* + ENX vs. Δ*ahslyA* and Δ*ahslyA* + ENX vs. WT + ENX have 14 common altered proteins, of which seven have the same protein expression trend and another seven have the opposite expression (Supplementary Table 1 and Figure 2E).

### Bioinformatics Analysis of Altered Proteins in Δ*ahslyA* + ENX/Δ*ahslyA* and Δ*ahslyA* + ENX/WT + ENX Comparisons

**FIGURE 3:**
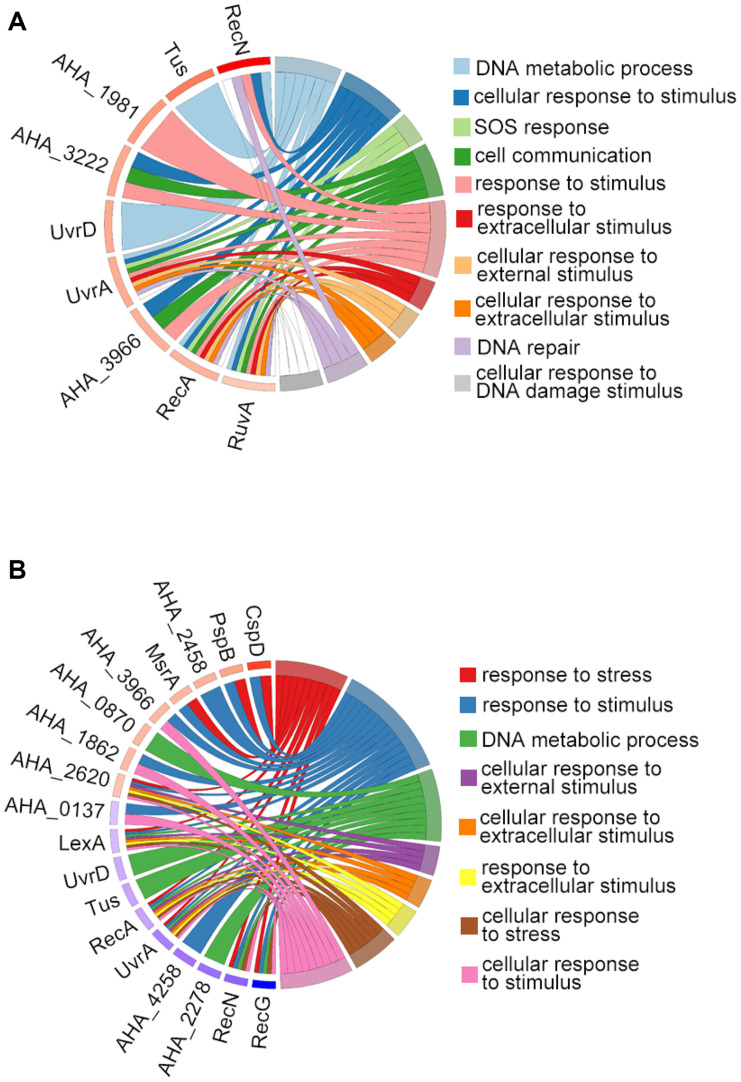
Chord plot showing GO terms BP analysis of differentially expressed proteins in the two comparisons. **(A)** Δ*ahslyA* + ENX vs. WT + ENX, and **(B)** Δ*ahslyA* + ENX vs. Δ*ahslyA*.

We then used GO terms enrichment to analyze altered proteins in both comparisons. In the biological process (BP) enrichment, the DNA metabolic process, cellular response to stimulus, SOS response, and cell communication were the most enriched terms in the Δ*ahslyA* + ENX vs. Δ*ahslyA* comparison. Additionally, the response to stress, the response to stimulus, the DNA metabolic process, and the cellular response to external stimulus were the most enriched terms in the Δ*ahslyA* + ENX vs. WT + ENX comparison. When comparing both group, the response to stress and the cellular response to stress in the Δ*ahslyA* + ENX vs. WT + ENX comparison changed significantly, while no change were observed in the Δ*ahslyA* + ENX vs. Δ*ahslyA* comparison (Figures 3A,B).

**FIGURE 4:**
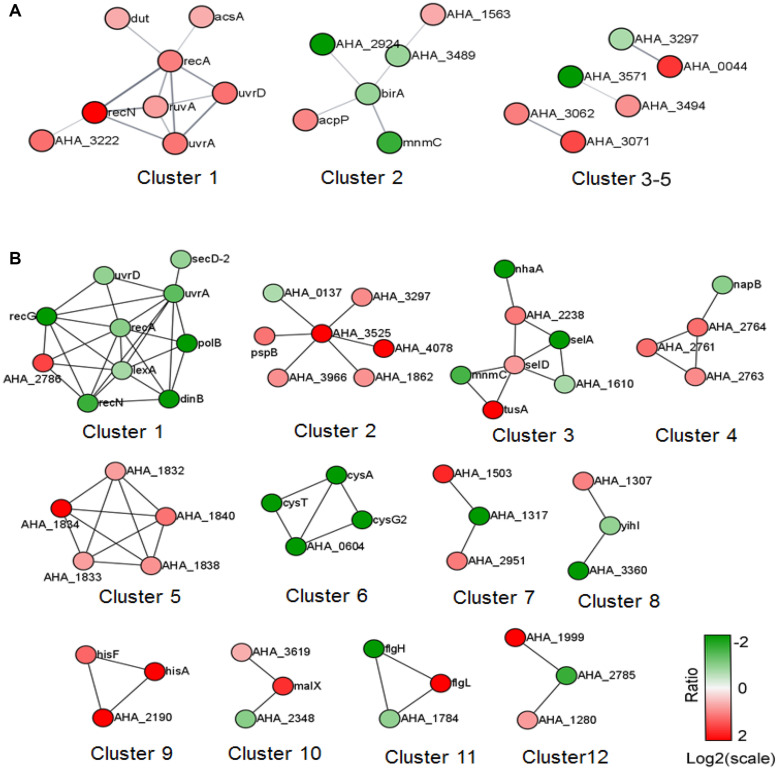
The PPI prediction network of altered proteins in the two comparisons. **(A)** Δ*ahslyA* + ENX vs. WT + ENX, and **(B)** Δ*ahslyA* + ENX vs. Δ*ahslyA*. The green dots represent downregulated proteins and red dots represent upregulated proteins.

We further analyzed the predicted PPI network of altered proteins in both comparisons using STRING software and then clustered them using the MCL algorithm. In the Δ*ahslyA* + ENX vs. Δ*ahslyA* comparison, there were five clusters that were enriched (Figure 4). Eight increasing proteins were clustered in cluster 1, and most of them were SOS or DNA repair-related proteins. There were six metabolic pathway-related proteins clustered in cluster 2, including three proteins that might be involved in acyl-CoA metabolism, namely, acyl-CoA thioester hydrolase YciA (gene name *AHA_1563*), acyl-CoA thioesterase I (*AHA_3489*), and acyl carrier protein (*acpP*). Both *AHA_3297* and *AHA_0044* are a sensor histidine kinase and sensory box/GGDEF family gene, respectively, which were present in clusters 3–5. It indicates that *ahslyA* may affect a bacterial two-component or cyclic di-GMP signaling system. The top 14 clusters of the Δ*ahslyA* + ENX vs. WT + ENX comparison are shown in Figure 3B. In cluster 1, at least seven proteins, including RecA, UvrA, UvrD, LexA, DinB, RecN, and RecG are involved in DNA repair processes and all seven were decreasing in abundance. *AHA_3525* is a response regulator, which was the network hub in cluster 2. Moreover, *AHA_3525* interacted with three TRs, namely, *AHA_0137* (response regulator, GltR), *AHA_1862* (response regulator protein), and *AHA_3966* (DNA-binding response regulator), in addition to *AHA_3297* (diguanylate cyclase DosC), and most of these genes were increasing in abundance. In cluster 3, most of altered proteins were metal ion-related proteins. For example, SelA, SelD, and the SelT/SelW/SelH domain (*AHA_1610*) were involved in selenium metabolism in prokaryotes. Additionally, clusters 4–6 were mostly clustered in oxidative respiration, uncharacterized protein and sulfate metabolism.

### The ENX-Resistance Capabilities of Proteins Regulated by *ahslyA*

**FIGURE 5:**
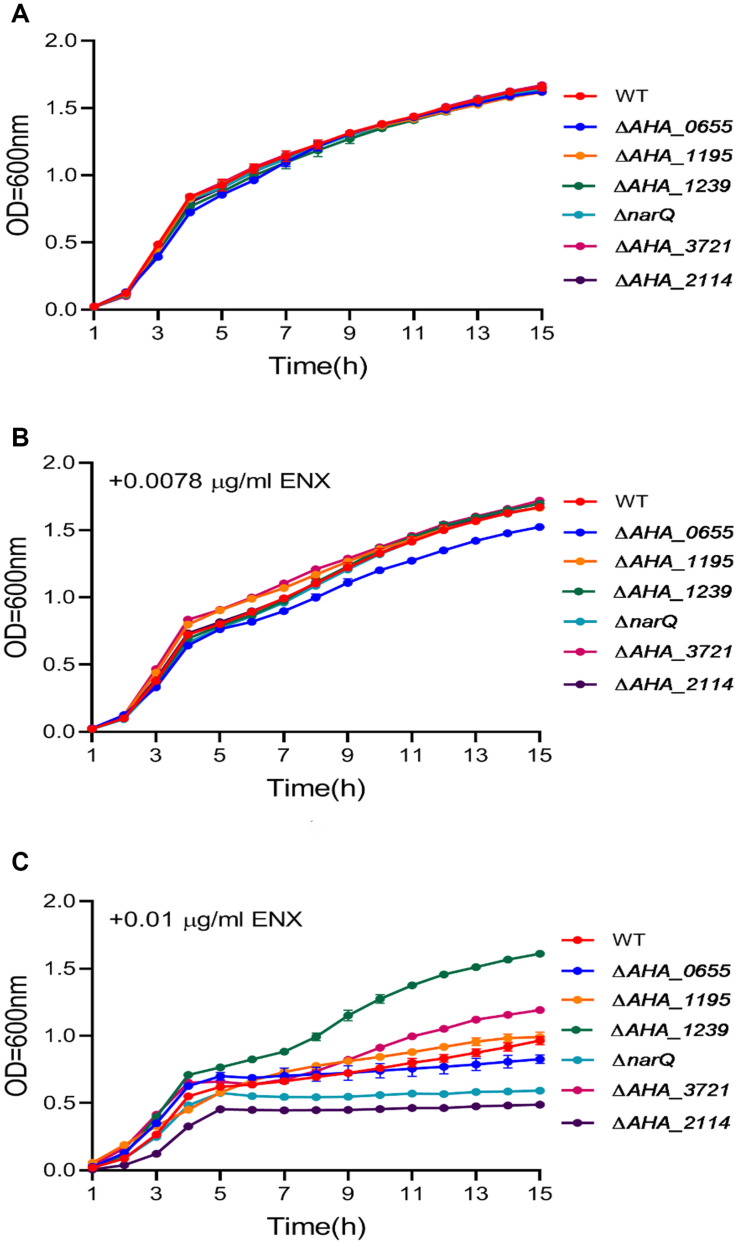
The Growth curves of *A. hydrophila* WT and a total six gene deletion strains with or without ENX treatment. **(A)** LB medium alone; **(B)** LB medium + 0.0078 μg/mL ENX; **(C)** LB medium + 0.01 μg/mL ENX.

In order to better understand the ENX resistance mechanism mediated by the TR *ahslyA*, we further assessed the effect of several gene mutants on the resistance to ENX, which were shown to be regulated directly or indirectly by *ahslyA* in our proteomics results. Selected gene deletion strains, including three genes related to decreasing in protein abundance (*AHA_0655*, *AHA_1195*, and *AHA_3721*) and three genes related to increasing in protein abundance (*AHA_1239*, *AHA_2114*, and *narQ*) in the Δ*ahslyA* + ENX vs. WT + ENX proteomic comparison, were successfully constructed *via* a two-step homologous recombination method using the primer pairs listed in Supplementary Table 2. Each mutant was assessed for sensitivity to ENX using an antibiotic susceptibility assay. As showed in Figure 5, the Δ*AHA_0655* exhibited a slightly decreased resistance while other mutants showed no significant difference to 0.0078 μg/mL of ENX. The Δ*AHA_2114* and Δ*narQ* showed a slight decrease in resistance to 0.01 μg/mL of ENX, and the Δ*AHA_1239* and Δ*AHA_3721* demonstrated significantly increased resistance to 0.01 μg/mL of ENX. These results suggested that *ahslyA* may regulate the transcription of *AHA_0655*, *narQ*, *AHA_3721*, *AHA_2114*, or *AHA_1239* to against ENX stress.

## Discussion

It is well known that bacterial TRs play a crucial regulatory role in diverse physiological and pathological functions. However, as there are hundreds of TRs are identified in prokaryotes and only a few of them have been well described, the intrinsic regulatory mechanisms of prokaryotic TRs are largely unknown (Brown et al., 2003). SlyA belongs to the TR MarR family that possesses a winged-helix DNA binding domain (Wilkinson and Grove, 2006). It was first reported to regulate virulence in many pathogens, such as *Salmonella typhimurium*, *Enterococcus faecalis*, and *Dickeya dadantii* (Libby et al., 1994; Haque et al., 2009; Michaux et al., 2011b). It has also been shown to play important roles in oxidative stress, bile salt stress, antimicrobial peptide resistance, heat, and acid stress (Buchmeier et al., 1997; Spory et al., 2002; Shi et al., 2004; Michaux et al., 2011a). Therefore, it suggests that this TR plays an important role in numerous physiological functions. However, the mechanism underlying these biological functions remains elusive.

Since SlyA plays an important role in the resistance to environmental stresses, we speculated that it may contribute to bacterial antibiotic resistance, because antibiotics are toxic small molecules like bile salts. To test our hypothesis, the antibiotic susceptibilities of the *slyA* gene deletion mutant and its complemented strains were analyzed in this study. We found that the Δ*ahslyA* had increased resistance against several quinolone antibiotics, suggesting that this TR may negatively regulate resistance to certain antibiotics, especially ENX. We then performed label-free quantitative MS to characterize the effect of *ahslyA* gene deletion on the proteome of *A. hydrophila* cells with or without ENX treatment. Here, we analyzed two group comparisons in order to understand the ENX resistance mediated by *ahslyA*. In the Δ*ahslyA* + ENX vs. Δ*ahslyA*, we identified 49 altered proteins, and when Δ*ahslyA* + ENX was compared to WT + ENX, there were 172 differentially expressed proteins. GO bioinformatics analysis showed that both comparisons were related to a stress response or stimulus, which indicated that *ahslyA* may play an important role in stress resistance to environmental factors, including antibiotics. Although, we have not obtained a significant KEGG (Kyoto Encyclopedia of genes and genes) pathway enriched altered proteins in either of the comparisons in this study. The PPI prediction plus the MCL algorithm showed that these altered proteins can be classified into several clusters. In the Δ*ahslyA* + ENX vs. Δ*ahslyA* comparison, five DNA repair-related proteins (RecA, RecN, RuvA, UvrA, and UrvD) were significantly increased in abundance. It is well known that quinolone antibiotics inhibit DNA synthesis and cause DNA strand cleavage or cell death. The underlying mechanism of quinolone antibiotic resistance in bacteria is due to the upregulation of DNA repair related proteins. In our previous study, 11 SOS responses or DNA repair-related proteins of *A. hydrophila* were reported to be increased under ENX stress (Zhang L.S. et al., 2020). Moreover, the deletion of *uvrA* decreased the ENX tolerance in *A. hydrophila*, which suggests the important role of the DNA repair process in protecting the DNA from quinolone-induced damage. Interestingly, we found that many of the DNA repair-related proteins decreased in abundance in the Δ*ahslyA* + ENX vs. WT + ENX comparison. This could have happened for a few reasons. First, the loss of *ahslyA* likely slowed down the toxic effect of ENX, so that bacteria did not trigger the DNA repair system. Second, the DNA repair response should be a last resort against ENX, as it may cause genetic mutations that influence bacterial survival. Third, *ahslyA* may regulate other drug resistance genes, aside from just DNA repair processes.

In both proteomic comparisons, the Δ*ahslyA* + ENX vs. WT + ENX comparison may be better than the Δ*ahslyA* + ENX vs. Δ*ahslyA* comparison to interpret *ahslyA*-mediated ENX resistance, because the first comparison is on the same ENX background. Therefore, we focused more on the properties of altered proteins between Δ*ahslyA* + ENX compared to Δ*ahslyA.* Of these altered proteins, we found *ahslyA* could directly or indirectly regulate at least 11 TRs (*gltR*, *yidZ*, *ycaN*, *citA*, *AHA_3297*, *AHA_0117*, *AHA_3721*, *AHA_1240*, *AHA_4233*, *AHA_1862*, and *AHA_3966*), which indicating that these TRs may construct a complicated gene regulatory network to maintain the intracellular homeostasis during ENX stress. Of these TRs, *AHA_3966* is homologous with *E. coli ompR*, with an identity of 49%. The *ompR* gene belongs to a well-known two-component regulatory system and plays important roles in multiple physiological functions, including antibiotic resistance (Lin et al., 2012; Zhang M.M. et al., 2020). Moreover, both *AHA_1862* and *AHA_3297* coded diguanylate cyclase and *AHA_3525* coded phosphodiesterase that govern the cellular level of c-di-GMP, which acts as a unique bacterial second messenger to trigger various cellular responses, such as in motility, biofilm formation, and antibiotics resistance (De et al., 2008; Gupta et al., 2014). However, the relationship between *ahslyA* and c-di-GMP is not clear.

To further understand the effect of *ahslyA* on the regulation of ENX resistance-related proteins, six target genes (*AHA_0655*, *AHA_1195*, *AHA_3721*, *AHA_1239*, *AHA_2114*, and *narQ*) that encode altered proteins in the proteomics data were selected to construct targeted gene deletion strains to determine their roles in susceptibility against ENX. Among these selected genes or proteins, *AHA_0655* (A0KG12) encodes an ATP-binding cassette transporter that belongs to a multi-drug efflux transporter family and plays a crucial role in the uptake of nutritional or toxic substrates from the environment, including antibiotics (Pletzer et al., 2015; Tang et al., 2019b). The decreased expression of A0KG12 in this study suggests that *ahslyA* may negatively regulate this protein to uptake ENX into cells. *AHA_1239* encodes a HlyD family secretion protein, was the protein that increased most in abundance in our MS data. *AHA_1239* increased 3,899 folds in the Δ*ahslyA* + ENX vs. WT + ENX comparison. Interestingly, *AHA_1239* is the downstream gene of *ahslyA* (*AHA_1240*), and its amino acid sequence has 30% identity to multi-drug resistance protein MdtN in *E. coli*. Moreover, the deletion of *ahslyA* and exposure to ENX stress also caused an upregulation of A0KK37 (*AHA_2114*), which is the multi-drug resistance protein MdtK. Since both proteins are members of the MATE (multi-drug and toxic compound extrusion) protein family. Further, the overexpression of MdtK increases resistance to norfloxacin, doxorubicin, and acriflavine in *Salmonella enterica* serovar Typhimurium. More, the *ahslyA* may negatively regulate both MdtN and MdtK to obtain ENX resistance in this study (Nishino et al., 2006). Additionally, the TRs A0KHI5 (*AHA_1195, YcaN*) and A0KPG8 (*AHA_3721*), nitrate sensor protein, A0KIL7 (*narQ*), belong to a two-component regulatory system play important roles on diverse biological functions in other bacterial species (Oshima et al., 2002). In this study, A0KHI5 and A0KPG8 were down-regulated, while NarQ increased in the Δ*ahslyA* + ENX vs. WT + ENX proteomics data, suggesting that they may affect ENX resistance regulated by *ahslyA*.

To test our hypothesis, the antibiotic susceptibilities of these six gene deletion mutants were determined. Our results showed that the deletion of *AHA_0655* slightly decreased the growth of *A. hydrophila* under low dose of ENX stress. Further the deletion of *AHA_2114* and *narQ* significantly decreased bacterial growth under high dose of ENX stress, while the deletion of *AHA_3721* increased antibiotic susceptibility in *A. hydrophila*. Which suggested that *ahslyA* may regulate *AHA_0655*, *narQ*, *AHA_3721*, and *AHA_2114* for ENX resistance. Although *AHA_1239* may act as a multi-drug resistance protein, the deletion of *AHA_1239* caused significant resistance to ENX in this study. The inherent reason is unknown, but based on the fact that *AHA_1239* is the downstream neighbor of *ahslyA*, the deletion of *AHA_1239* may affect the expression of *ahslyA* and then trigger other ARGs or systems against ENX stress. Overall, our data demonstrated the important role of *ahslyA* in the multiple ARGs regulation during ENX resistance and provided novel insights into the effects of TRs on the antibiotic resistance of bacteria.

## Data Availability Statement

The datasets presented in this study can be found in online repositories. The names of the repository/repositories and accession number(s) can be found below: http://www.proteomexchange.org/, PXD024843.

## Author Contributions

XL and LZ conceptualized and validated the study. ZL, LL, and LZ performed the methodology and the data curation and wrote the manuscript for the final draft. ZL, GW, and QS performed the formal analysis. XL was responsible for the resources, supervised the study, and performed the funding acquisition. RS, WY, HT, and XL wrote, reviewed, and edited the manuscript. All authors contributed to the article and approved the submitted version.

## Conflict of Interest

The authors declare that the research was conducted in the absence of any commercial or financial relationships that could be construed as a potential conflict of interest.

## Footnotes

1 https://david.ncifcrf.gov/

2 https://string-db.org

3 http://www.cytoscape.org

## Supplementary Material

Supplementary Figure 1 Construction and confirmation of the six gene deletion strains.

Supplementary Table 1 The identification of 14 common altered proteins between both group comparisons using label-free analysis.

Supplementary Table 2 The primer pairs used in this study.
