# Supplementary material for: Proteomics Analysis Reveals Bacterial Antibiotics Resistance Mechanism Mediated by ahslyA Against Enoxacin in Aeromonas hydrophila
Source: Front Microbiol. 2021 Jun 8;12:699415. doi: 10.3389/fmicb.2021.699415 (PMC8217646; doi:10.3389/fmicb.2021.699415)
Supplement: Supplementary Figure 1 — Construction and confirmation of the six gene deletion strains. [file Data_Sheet_1.docx]

**Supplementary Figure S1**


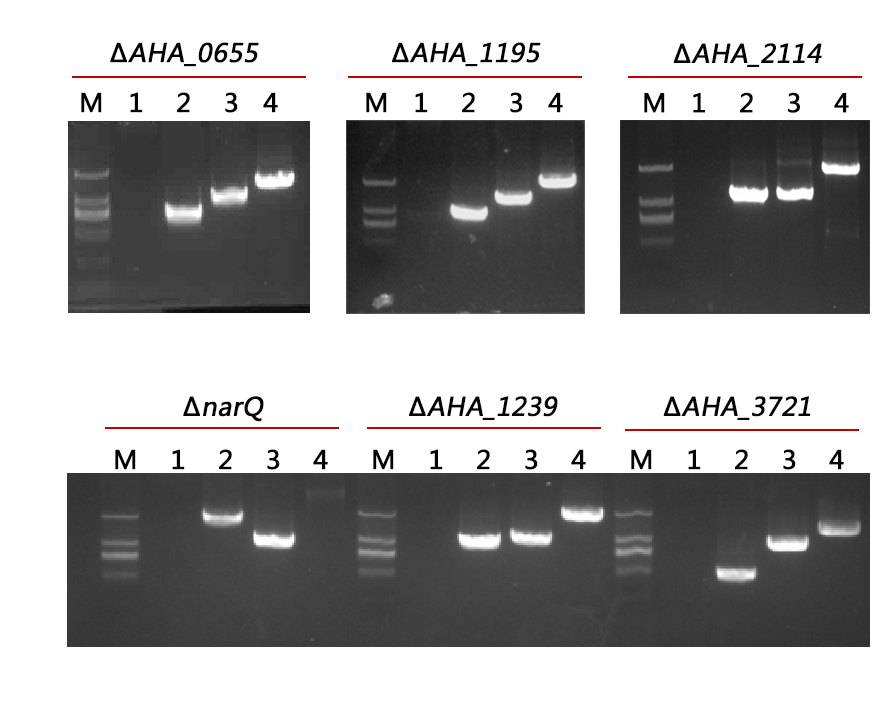


**Supplementary Figure S1. Construction and confirmation of the six gene deletion strains.** Fragments of target DNA amplified using the primer pair P5/P6 and P7/P8 in the target mutant and WT strain as controls are displayed. M: DL 2000 marker. Lane1-4: target deleted strain (P5/P6), WT (P5/P6), target deleted strain (P7/P8), WT (P7/P8), respectively.
