# Supplementary material for: Proteomics Analysis Reveals Bacterial Antibiotics Resistance Mechanism Mediated by ahslyA Against Enoxacin in Aeromonas hydrophila
Source: Front Microbiol. 2021 Jun 8;12:699415. doi: 10.3389/fmicb.2021.699415 (PMC8217646; doi:10.3389/fmicb.2021.699415)
Supplement: Supplementary Table 1 — The identification of 14 common altered proteins between both group comparisons using label-free analysis. [file Table_1.docx]

**Supplementary Table S1. The identification of 14 common altered proteins between both group comparisons using label-free analysis**

| Proteins ID | Gene ID | Protein description | log2(*ΔahslyA* + ENX/WT+ENX) | p-value | log2(Δ*ahslyA*+ ENX/Δ*ahslyA*) | p-value |
| --- | --- | --- | --- | --- | --- | --- |
| A0KN52 | AHA_3212 | Uncharacterized protein | 2.678371 | 0.007886 | 2.960262 | 0.004596 |
| A0KKT3 | AHA_2361 | Uncharacterized protein | 1.925743 | 0.044148 | 0.653175 | 0.049435 |
| A0KI65 | malX | PTS system, maltose and glucose-specific IIBC component | 1.617822 | 0.0134 | 0.800314 | 0.045968 |
| A0KMF4 | AHA_2951 | Uncharacterized protein | 1.015505 | 0.006795 | 1.603295 | 0.002324 |
| A0KND7 | AHA_3297 | Sensor histidine kinase | 0.871904 | 0.005936 | -0.61522 | 0.025654 |
| A0KQ49 | AHA_3966 | DNA-binding response regulator | 0.842521 | 0.043261 | 1.020062 | 0.031097 |
| A0KMR3 | AHA_3062 | Formate dehydrogenase iron-sulfur subunit | 0.776461 | 0.031619 | 0.977926 | 0.029424 |
| A0KET2 | uvrD | DNA helicase | -0.81417 | 0.003282 | 1.090236 | 0.004723 |
| A0KM73 | tus | DNA replication terminus site-binding protein | -0.88931 | 0.000299 | 1.641749 | 0.002522 |
| A0KPG3 | recA | Protein RecA | -0.89149 | 0.000317 | 0.982447 | 0.002058 |
| A0KQ38 | uvrA | UvrABC system protein A | -1.2485 | 0.00394 | 1.052292 | 0.01832 |
| A0KKK0 | AHA_2278 | Site-specific recombinase, phage integrase family | -1.4134 | 0.01114 | 4.634987 | 0.008861 |
| A0KJJ9 | mnmC | tRNA 5-methylaminomethyl-2-thiouridine biosynthesis bifunctional protein MnmC | -1.43805 | 0.049025 | -1.55761 | 0.032454 |
| A0KMI9 | recN | DNA repair protein RecN | -1.53537 | 0.000911 | 2.505596 | 0.00608 |
