# Supplementary material for: Proteomics Analysis Reveals Bacterial Antibiotics Resistance Mechanism Mediated by ahslyA Against Enoxacin in Aeromonas hydrophila
Source: Front Microbiol. 2021 Jun 8;12:699415. doi: 10.3389/fmicb.2021.699415 (PMC8217646; doi:10.3389/fmicb.2021.699415)
Supplement: Supplementary Table 2 — The primer pairs used in this study. [file Table_2.docx]

**Supplementary Table S2. The primer pairs used in this study.**

| Gene | oligonucleotide sequence 5′-3 | Purpose |
| --- | --- | --- |
| *AHA_0655*-P1  *AHA_0655*-P2  *AHA_0655*-P3  *AHA_0655*-P4  *AHA_0655*-P5  *AHA_0655*-P6  *AHA_0655*-P7  *AHA_0655*-P8 | CGATCCCAAGCTTCTTCTAGATGGTGTGGATCACCACCAGG  CAGCTTCCTTCGTCCTGATTCTAACGCTGGC  AATCAGGACGTCTGTTGCTGGTCGCCGC  CATGAATTCCCGGGAGAGCTCTCTGGTAGCTGGCGTAAGGTACG  CATGAATTCCCGGGAGAGCTCTCTGGTAGCTGGCGTAAGGTACG  TCAATGCATCAAGAATTCGGC  TGATGAAAATGGTGTCGTCACC  TAACGGCATACTCCTTGTG | Gene deletion  Gene deletion  Gene deletion  Gene deletion  Gene deletion  Gene deletion  Gene deletion  Gene deletion |
| *AHA_1195*-P1  *AHA_1195*-P2  *AHA_1195*-P3  *AHA_1195*-P4  *AHA_1195*-P5  *AHA_1195*-P6  *AHA_1195*-P7  *AHA_1195*-P8 | CATGAATTCCCGGGAGAGCTCCTTCTACTGCAATGTTCATGTCAGAC  CTGGTAGTGTGTTGAGCAACAGGGTACGTCAGC  GTTGCTCAACACACTACCAGCCACGCTGATTG  CGATCCCAAGCTTCTTCTAGATGTCGGTACCCTGTTCAATGC  TTAATATGGCCATCACCCGTAC  GGTAGTGTCCCTTGAGCAT  TTGATCGGCAGGAAGAGTAC  CCCACCATGATGTAGTTGAG | Gene deletion  Gene deletion  Gene deletion  Gene deletion  Gene deletion  Gene deletion  Gene deletion  Gene deletion |
| *AHA_1239*-P1  *AHA_1239*-P2  *AHA_1239*-P3  *AHA_1239*-P4  *AHA_1239*-P5  *AHA_1239*-P6  *AHA_1239*-P7  *AHA_1239*-P8 | CATGAATTCCCGGGAGAGCTCGCCTGCTAAACAACTACAATGCTT  TCACCCACTCTCTCTCTTCACTCTGTGCAC  TGAAGAGAGAGAGTGGGTGACGGCAATGACTTTGTG  TGAAGAGAGAGAGTGGGTGACGGCAATGACTTTGTG  ATGACCCCGGATCAACAATTC  TCAGTAGATGTAGTGCAGCAG  GCTGCCAACAGGACATTCAAAC  TCCCGCAGATCGAAGATCTG | Gene deletion  Gene deletion  Gene deletion  Gene deletion  Gene deletion  Gene deletion  Gene deletion  Gene deletion |
| *narQ*-P1  *narQ*-P2  *narQ*-P3  *narQ*-P4  *narQ*-P5  *narQ*-P6  *narQ*-P7  *narQ*-P8 | CGATCCCAAGCTTCTTCTAGAGATAACCATTGGTGGTCATGGC  TCTCTCTTCCTTCACTTCAGACAATAACC  CTGAAGTGAAGGAAGAGAGAAATATAGTGTTCTGGTCGTGGATG  CATGAATTCCCGGGAGAGCTCAAATATGGAGTACGGAGGCGAC  AGTGGTTCAAGGTACATTCG  TCAGGCATCTCTTGTTAGGCTG  GTTCTCACCGGTGATTTGGT  CTGCTCCAGGTACATGACAG | Gene deletion  Gene deletion  Gene deletion  Gene deletion  Gene deletion  Gene deletion  Gene deletion  Gene deletion |
| *AHA_3721*-P1  *AHA_3721*-P2  *AHA_3721*-P3  *AHA_3721*-P4  *AHA_3721*-P5  *AHA_3721*-P6  *AHA_3721*-P7  *AHA_3721*-P8 | CATGAATTCCCGGGAGAGCTCAGATTGGAAATGCCGCTGG  ATCCCACTCTCCGCCTAATTCGTCACAAGG  AATTAGGCGGAGAGTGGGATCACTCTTCCAGGC  CGATCCCAAGCTTCTTCTAGAATGCGAAGGCATTTCGCC  ATGATGAAGTGCTGGTCGC  GAAGAGTGATCCCACTCTCC  TGACCGAAGGCTGGCCATT  CAACAGACCGTCTACACCCTG | Gene deletion  Gene deletion  Gene deletion  Gene deletion  Gene deletion  Gene deletion  Gene deletion  Gene deletion |
| *AHA_2114*-P1  *AHA_2114*-P2  *AHA_2114*-P3  *AHA_2114*-P4  *AHA_2114*-P5  *AHA_2114*-P6  *AHA_2114*-P7  *AHA_2114*-P8 | CGATCCCAAGCTTCTTCTAGAGTACTATGCCGGTGAACATGTCTC  CTGTTGTGACACCTCCGAGTGGATCAG  ACTCGGAGGTGTCACAACAGGCTGCGCAAGAAC  CATGAATTCCCGGGAGAGCTCGACATCGCCTACGCCATGTC  GTGCAACGTTATTGGTCCGAG  GAGTTTGCCCTATTGCGCCTT  CAGATCGAAGCTGACCAGCTC  TCTGATTGCAGGGGAGTGGTG | Gene deletion  Gene deletion  Gene deletion  Gene deletion  Gene deletion  Gene deletion  Gene deletion  Gene deletion |

**Remarks:**

P1 and P2 primers were used to amplify the upstream fragment of the target gene; P3 and P4 primers were used to amplify the downstream fragment of the target gene; P5 and P6 primers were used to amplify the target gene fragment or the internal part of the target gene; P7 and P8 primers were used to amplify the upstream and downstream fragments which extended about 100 bp to both sides, and P5 and P6, P7 and P8 were used to verify whether the target gene was knocked out correctly.
